# Impact of an external ventricular shunt (EVD) handling protocol on secondary meningitis rates: a historical cohort study with propensity score matching

**DOI:** 10.1186/s12883-023-03080-2

**Published:** 2023-01-23

**Authors:** Daphna Hoefnagel, Victor Volovici, Ellianne J. dos Santos Rubio, Anne F. Voor in’t Holt, Clemens M. F. Dirven, Margreet C. Vos, Ruben Dammers

**Affiliations:** 1 Department of Neurosurgery, Erasmus MC Stroke Center, Erasmus MC University Medical Center, Doctor Molewaterplein 40 Room-#: Na-2112, 3015 GD Rotterdam, the Netherlands; 2 Department of Public Health, Center for Medical Decision Making, Erasmus MC University Medical Center, Rotterdam, the Netherlands; 3 Department of Neurosurgery, St. Elisabeth Hospital, Willemstad, Curaçao; 4 Department of Medical Microbiology and Infectious Diseases, Erasmus MC University Medical Center, Rotterdam, the Netherlands

**Keywords:** External ventricular drainage, EVD handling protocol, Meningitis, Ventriculitis, Central nervous system infection, Protocol, Quality improvement

## Abstract

**Background:**

External ventricular drainage (EVD) is frequently used in neurosurgical procedures for cerebrospinal fluid (CSF) drainage. It is, however, associated with high infection rates, namely secondary meningitis and ventriculitis. Based on a previous high prevalence of these infections among patients with EVDs, we have proposed and implemented a protocol in an effort to decrease the infection rate. The aim of this study was to measure the effect of hospital-wide implementation of the EVD handling protocol on secondary EVD infections.

**Patients and methods:**

We included 409 consecutive patients who received a new EVD for other indications than infectious pathologies from January 2000 until June 2012. Patients above 18 years of age were divided into pre- (*n* = 228) and post-protocol (*n* = 181) groups. Patient and disease demographics, as well as EVD data together with confounders for secondary meningitis were recorded in a database. Propensity score matching was then performed to create groups matched for sex, age, reason for drainage, type of shunt, time in situ and duration of surgery to place the EVD. Binomial logistic regression for confounder adjustment and regression discontinuity analyses were then performed on the matched cohort.

**Results:**

Infections occurred more frequently in the pre-protocol group (23% vs 9%, *p* <  0.001). The incidence of infection was 33/1000 drain-days pre-protocol and 9/1000 drain-days post-protocol. Regression analysis in a propensity score-matched cohort (*n* = 103 in the pre- and *n* = 178 in the post-protocol groups) showed that the pre-protocol period was independently associated with more infections (OR 2.69; 95%-CI 1.22–5.95, *p* = 0.01).

**Conclusions:**

The incidence of secondary EVD infections can be reduced significantly by the implementation of a strict hospital-wide EVD handling protocol.

## Introduction

Ventriculostomy with an external ventricular drain (EVD), is a common neurosurgical procedure to relieve and/or monitor increased intracranial pressure through constant or intermittent cerebrospinal fluid (CSF) drainage. Secondary meningitis and/or ventriculitis are well-known complications and account for a high morbidity and mortality [1]. Additionally, these infections are associated with longer intensive care unit and hospital stay, and increased health care costs [2, 3]. EVD infection rate has been reported to occur in as low as 0%, and in as high as 52% of patients [4–6].

Many methods to prevent EVD infections are available, from more general measures, such as rigorous disinfection of the skin, pre- and peroperative prophylaxis and shortening the duration of the procedure. Others are more specific, such as, antibiotic impregnated shunts [2], prolonged prophylactic antibiotics [3], or combinations of these in protocols [4, 7, 8]. Obviously, mere individual application of these measures may not be sufficient and a protocol may be required. In recent years, many such protocols have been published [3, 4, 6–10] resulting in rates as low as 0% after implementation being reported [4].

In an earlier paper, we have audited our practice and reported a high incidence rate of EVD infection, 23.2% [1]. Our study aim was to test the hypothesis that in this before –after intervention study, the implementation of a hospital-wide EVD protocol would result in fewer EVD infections.

## Methods

This study was conducted in the Erasmus MC University Medical Center in Rotterdam, the Netherlands (Erasmus MC), a tertiary, academic, level I trauma center. We designed a quasi-experimental temporal study: a prospective observational cohort was compared with historical controls, of which data were derived from the patient record system and stored in a separate database. This database was also used for our previous study and updated prospectively [1]. The pre-protocol group included patients with an EVD from January 2000 until April 2005 and the post-protocol group included patients with an EVD from January 2007 until June 2012. The study was deemed IRB exempt by the medical ethics committee of the Erasmus MC.

Patient demographics, underlying diagnosis, the use of prophylactic antibiotics, type of drainage (Rickham reservoir or EVD), frequency of CSF sampling, irrigation, and CSF leakage, period of drainage, as well as the outcome secondary infection were recorded. Two investigators (DH an EdSR) collected all data consistent with the criteria proposed. The exclusion criteria were: 1) age < 18 years, 2) pre-existent CSF infection.

### EVD surgical protocol

Placement of the ventriculostomy follows standard EVD protocol placement. Thirty minutes prior to incision, all patients receive either a single intravenous dose of 2 g Cefazolin or, in case of an allergy, 600 mg of Clindamycin or 1000 mg flucloxacilline. After liberal hair removal through clipping and rigorous disinfection and draping with standard sterile techniques, a skin incision is made centered around Kocher’s point. After burr hole placement, the dura is opened using monopolar coagulation. Depending on the underlying diagnosis and attending neurosurgeon, a subcutaneously tunneled EVD (at least 5 cm from the surgical insertion site) or a Rickham reservoir for percutaneous CSF drainage is inserted. In more recent years, tunneled EVDs represent the norm and Rickham reservoirs are largely abandoned, save for very specific cases.

For a subcutaneously tunneled EVD we use a linear incision. After obtaining CSF, the catheter would be then tunneled 5–6 cm from the insertion site and connected to an external CSF drainage system *(Duet External Drainage and Monitoring System, SmartSite Injection Sites*, Medtronic Inc., Minneapolis, MN, USA). The catheter is sutured to the skin to prevent dislodgment and a sterile nonocclusive dressing was applied. In case of a Rickham reservoir with percutaneous drainage a semicircular incision would be used. After skin closure, the Rickham reservoir would be pierced with a winged infusion needle (either a 21-gauge Venofix set, Braun Medical Industries, Melsungen, Germany or a *BD Value-set, Becton, Franklin Lakes, NJ, USA)*, and subsequently connected to an external CSF drainage system.

### Intervention: post-operative EVD handling protocol

The intervention constituted of a care bundle (Table 1), that addressed different aspects of post-operative EVD care: head dressing, frequency of CSF sampling, handling of CSF sampling, treatment of meningitis and EVD flushing.

**Table 1:** The intervention bundle and differences when compared to the pre-protocol phase

Area of intervention	Pre-protocol	Post-protocol
Head dressing	No formal recommendation	Sterile dressing as long as EVD remains in situ, changed every 72 hours, except for cases where EVD malfunction or hemorrhage is suspected.
CSF tapping	Daily CSF tapping	Only when all other infection foci have been excluded and there are clinical symptoms that suggest meningitis
Handling of CSF tapping	No formal recommendation	Sterile gloves, multiple disinfection moments before tapping
Meningitis treatment strategy	No formal recommendation	Multidisciplinary meetings with infectiologists
EVD flushing	No formal recommendation	Use of sterile gloves, multiple disinfection moments

Cerebrospinal fluid (CSF) would be sampled according to a written protocol, at the time of insertion, when infection is suspected, 48–72 hours after initiation of antibiotic treatment, and upon removal of the EVD.

In the pre-protocol period CSF would be routinely sampled three times a week. In the post-protocol era, CSF would only be collected in case meningitis was strongly suspected based on clinical symptoms and other infection foci had been excluded.

The CSF samples would be collected via the proximal 3-way needleless stopcock by a neurosurgical trainee or physician assistant following strict aseptic measures. The rubber sealed cap would be disinfected with alcohol 70% and a total of 5 mL CSF would be sampled and sent to the medical microbiology laboratory for Gram’s stain, culture, cell count, and for chemical analyses on glucose, and protein concentration. Catheters were left in place if clinically indicated and changed only if they malfunctioned or in cases of highly potent infections. Infection treatment strategies were discussed regularly during multidisciplinary meetings with the infectious disease specialists. Drain blockage would usually be resolved by flushing the drain with 2 mL sterile 0.9% NaCl, following the same aseptic protocol.

### Definition of EVD secondary infection

Infection was defined according to the Centers for Disease Control and Prevention (CDC) criteria [11], as a positive CSF culture on the day that the sample was obtained and at least two symptoms of meningitis. The term contamination was used when a patient had only one positive CSF culture for a common skin pathogen, the results of consecutive samples were negative, and no treatment had been started.

### Statistical analysis and propensity score matching

All patient and disease demographics, as well as EVD data (CSF leakage, infection, frequency of CSF sampling, and number of catheter days) were evaluated. Continuous values were expressed as median ± interquartile range (IQR) and compared using analysis of variance and Student-t test. Non-parametric data were compared using χ^2^ tests or the Mann Whitney U test. Significant (*p* <  0.01) associations identified by univariate analysis were further assessed by binomial logistic regression analysis to determine the independent predictors of an EVD related infection. Furthermore, propensity score matching was used to create two groups balanced for the confounders we measured in the two groups. The two groups were matched for sex, age, reason for drainage, type of shunt, time in situ and duration of surgery to place the EVD. As we did not have a sample size large enough to easily create balanced groups, we chose a wider caliper, equal to 0.25 times the standard deviation of the propensity score. Furthermore, to minimize the mean squared error we subsequently used a 1000 sample bootstrapped binomial logistic regression model for confounder adjustment on the matched cohort. Results of these associations were expressed as odds ratios (OR) and their 95% confidence intervals (95%-CI). A *p*-value < 0.05 was considered significant.

## Results

We included a total of 409 patients, of which the pre-protocol group consisted of 228 patients and the post-protocol group of 181 patients.

EVDs were mainly placed in the context of emergency surgery (Tables 2 and 3) in both the pre- and post-protocol period. In total, there were 53/228 (23%) EVD-related infections in the pre-protocol patient group and 16/181 (9%) EVD-related infections in the post-protocol patient group, respectively (*p* <  0.001). After ventriculostomy placement, the infection was diagnosed, 8.0 ± 9.0 (mean + −SD) days for the pre-protocol group and 9.5 ± 10.0 days for the post-protocol group. In the pre-protocol period 82% of patients received Flucloxacillin or amoxicillin/clavulanic acid as perioperative prophylaxis, compared to only 2% of patients in the post-protocol period. Patients in the post-protocol era were administered Cefazolin as perioperative prophylaxis in 95% of cases, compared to only 13% in the patients of the pre-protocol group.

**Table 2:** Patient demographics and EVD (external ventricular drain) catheter data for the entire cohort (*n* = 409)

	Pre-protocol *n* = 228	Post-protocol *n* = 181	*p* -value
*Patient factors*			
Sex: Male (%)	50	44	0.31
Age (year; median [IQR])	60 [49–70	56 [47–66]	**0.006**
Emergency surgery (%)	95	86	**< 0.001**
Indication for EVD (%)			**< 0.001**
SAH/IVH	48	60	
Tumor	10	12	
Trauma	3	9	
Other^a^	39	19	
*Catheter factors*			
Surgeon			**< 0.001**
PGY 1–3	26	39	
PGY 4–6	35	18	
Neurosurgeon	39	43	
Tunnelled EVD^b^ (%)	47	80	**< 0.001**
Operating time (min; median [IQR])	66 [54–80]	73 [58. 5–96]	**0.005**
Prophylactic antibiotics (%)	38	86	**< 0.001**
CSF leakage (%)	10	13	0.52
Frequency of CSF sampling (median ± IQR)	1 [1–3]	1 [1–2]	0.07
Duration of drainage (days; median ± IQR)	5.5 [2–12]	7 [3–12]	0.11

Shown are median values ± interquartile ranges (IQR). The percentage between brackets is the percentage of patients within that group. *P*-values represent the results of univariate analysis of the respective parameter. A *p*-value of < 0.05 was considered significant

*CSF* cerebral spinal fluid, *EVD* external ventricular drainage, *IVH* intraventricular hematoma, *SAH* subarachnoid hemorrhage, *PGY* post graduate year of the surgeon, *Min* minutes

^a^includes infratentorial intracerebral hematoma (38.4%), supratentorial intracerebral hematoma (23.3%), cerebellar infarction (17.8%), colloid cyst (5.5%), ventriculoperitoneal shunt malfunction (4.1%), and miscellaneous (11.0%)

^b^the remaining percentage received a Rickham reservoir

**Table 3:** Patient demographics and EVD (external ventricular drain) catheter data for the matched cohort (*n* = 281)

	Pre-protocol *n* = 103	Post-protocol *n* = 178	*p* -value
*Patient factors*			
Sex: Male (%)	50	44	0.32
Age (year; median [IQR])	59 [49–69]	56 [47–66]	0.30
Emergency surgery (%)	100	85	**< 0.001**
Indication for EVD (%)			**0.01**
SAH/IVH	50	60	
Tumor	13	12	
Trauma	3	9	
Other^a^	34	19	
*Catheter factors*			
Surgeon			0.11
PGY 1–3	35	40	
PGY 4–6	27	16	
Neurosurgeon	38	44	
Tunnelled EVD^b^ (%)	57	81	**< 0.001**
Operating time (minutes; median [IQR])	69 [54–82]	73 [58–94]	0.053
Prophylactic antibiotics (%)	49	86	**< 0.001**
CSF leakage (%)	9	13	0.35
Frequency of CSF sampling (median [IQR])	1 [0–3]	1 [0–2]	0.07
Duration of drainage (days; median [IQR])	5 [2–12]	7 [3–12]	0.13

Shown are median values ± interquartile ranges (IQR). The percentage between brackets is the percentage of patients within that group. *P*-values represent the results of univariate analysis of the respective parameter. A *p*-value of < 0.05 was considered significant

*CSF* cerebral spinal fluid, *EVD* external ventricular drainage, *IVH* intraventricular hematoma, *SAH* subarachnoid hemorrhage, *PGY* post graduate year of the surgeon, *Min* minutes

^a^includes infratentorial intracerebral hematoma (38.4%), supratentorial intracerebral hematoma (23.3%), cerebellar infarction (17.8%), colloid cyst (5.5%), ventriculoperitoneal shunt malfunction (4.1%), and miscellaneous (11.0%)

^b^the remaining percentage received a Rickham reservoir

The most common bacteria found in cultures taken from patients with secondary infected EVDs were coagulase negative staphylococci. The prevalence of contaminated samples was not significantly different between the pre- and post-protocol groups (*p* = 0.36).

Using a regression discontinuity design and binomial logistic regression analysis, before and after propensity score matching (Tables 4 and 5), factors significantly associated with EVD infections were the pre-protocol phase, the presence of a CSF fistula, and the number of times CSF was sampled.

**Table 4:** Binomial logistic regression model with covariates associated with the risk of an EVD-related infection (*n* = 409, entire cohort, bootstrapped with 1000 samples)

	OR	95%-CI	*p* -value
CSF fistula	2.13	0.88–5.16	0.09
No Protocol	2.69	1.22–5.95	**0.01**
Junior resident performing surgery	0.87	0.39–1.96	0.75
Senior resident performing surgery	0.76	0.39–1.96	0.51
Staff member performing surgery	Reference category		
Times CSF sampled	1.55	1.28–1.88	**< 0.0001**
Duration of drainage	1.03	0.98–1.08	0.21
EVD placement for SAH	0.50	0.25–1.09	0.08
EVD placement for tumor	0.25	0.19–18.52	0.25
EVD placement for other types of hydrocephalus	Reference category		

*OR* odds ratio, and *95%-CI* 95 percentage confidence interval, *CSF* cerebrospinal fluid, *EVD* external ventricular drainage, *SAH* subarachnoid hemorrhage

**Table 5:** Binomial logistic regression results applied to the propensity score- matched cohort (*n* = 281, with bootstrapping)

	*p*-value	OR	95% Confidence Interval
Noprotocol	**0.008**	3.37	1.36–8.29 - 0.61
CSF fistula	**0.03**	3.32	1.12–9.81 -5.86
Duration of drainage	0.90	1.00	0.93–1.07
Number of times CSF sampled	< 0.001	1.63	1.25–2.13
Junior resident performing surgery	0.98	0.99	0.38–2.54
Senior resident performing surgery	0.86	1.10	0.37–3.21
Staff member performing surgery	Reference category		

## Discussion

In a quasi-experimental design, using a before-and-after cohort, we showed a reduction of the odds for secondary meningitis, with the period before use of handling protocol being significantly associated with a higher odds of secondary meningitis.

### Infection rate

Compared to our previous retrospective single center study [1] the incidence of EVD related infection is decreased from 23 to 9% with the use of a strict EVD handling protocol. Various protocols and strategies are being used worldwide in an effort to reduce the prevalence of secondary meningitis [4, 7, 8].

It is quite difficult to point out exactly which of the interventions we have implemented as part of the bundle is the single most important one. Raising awareness to the possibility of contamination of the catheter system which can result in a secondary meningitis might be one of the main drivers of the reduced number of infections [12]. This issue is likely a major factor contributing to success, the so-called Hawthorne effect [13]. Simply raising awareness and measuring an outcome might lead to improvement.

We consider the reduction of the frequency of CSF sampling as an essential contribution to lowering the infection incidence rate. Indication to sample CSF is now restricted to cases for which meningitis is suspected based on clinical symptoms and all other infection foci have been ruled out. The number of times CSF was sampled was an independent risk factor for infection and thus should be avoided unless absolutely necessary as even sterile manipulation or dressing disruptions are a known risk factor for contamination of the catheter [14].

Healthcare-related infections have a deleterious effect on the postoperative course and may lead to serious morbidity and mortality of neurosurgical patients.

Despite being one of the “routine” neurosurgical procedures, the costs associated with developing secondary meningitis from an EVD are very high, estimated at around 2800 euro per infected patient in the Netherlands. From a health economics perspective, minimal interventions and changes in practice may lead to a large benefit, both for the patients and for the rising healthcare costs.

### Study limitations

Our study has the following limitations: the retrospective nature, which favors confounding by indication and missing values. Confounding by indication is always present in observational studies and in this respect, we have performed propensity score matching in order to try to achieve groups balanced with respects to measured confounders. Unmeasured confounding and the wide confidence intervals prevent us from making very definitive causal statements, but the direction of the effect and effect size are consistent between the unmatched and matched groups, strengthening our confidence in these results.

The intervention consists of a bundle, and we cannot be certain which parts of the bundle were essential for the reduction in the infection rates and which were not. We cannot definitively infer that the reduction is based on the protocol itself or the awareness it created. We also do not have full data on protocol adherence, but the neurosurgical trainees and PAs were the only professionals handling the EVDs and in this group adherence was > 90%, according to an internal 2013 audit.

## Conclusions

We showed a significant decrease in the incidence rate of EVD-related infections after the implementation of a hospital-wide EVD handling protocol. Further studies should seek to unravel the relative contributions of the bundle elements to risk reduction. Innovative ways to further decrease the risk of secondary EVD-related infections are needed.

## Data Availability

Data may be made available upon written reasonable request to the corresponding author.
